# A Strategy for Field Evaluations of Exposures and Respiratory Health of Workers at Small- to Medium-Sized Coffee Facilities

**DOI:** 10.3389/fpubh.2021.705225

**Published:** 2021-11-11

**Authors:** M. Abbas Virji, Kristin J. Cummings, Jean M. Cox-Ganser

**Affiliations:** Respiratory Health Division, Centers for Disease Control and Prevention, National Institute for Occupational Safety and Health, Morgantown, WV, United States

**Keywords:** data pooling, harmonization, coffee roasting and packaging, alpha-diketones, respiratory health, exposure assessment

## Abstract

Coffee production is a global industry with roasteries throughout the world. Workers in this industry are exposed to complex mixtures of gases, dusts, and vapors including carbon monoxide, carbon dioxide, coffee dust, allergens, alpha-diketones, and other volatile organic compounds (VOCs). Adverse respiratory health outcomes such as respiratory symptoms, reduced pulmonary function, asthma, and obliterative bronchiolitis can occur among exposed workers. In response to health hazard evaluations requests received from 17 small- to medium-sized coffee facilities across the United States, the National Institute for Occupational Safety and Health conducted investigations during 2016–2017 to understand the burden of respiratory abnormalities, exposure characteristics, relationships between exposures and respiratory effects, and opportunities for exposure mitigation. Full-shift, task-based, and instantaneous personal and area air samples for diacetyl, 2,3-pentanedione and other VOCs were collected, and engineering controls were evaluated. Medical evaluations included questionnaire, spirometry, impulse oscillometry, and fractional exhaled nitric oxide. Exposure and health assessments were conducted using standardized tools and approaches, which enabled pooling data for aggregate analysis. The pooled data provided a larger population to better address the requestors' concern of the effect of exposure to alpha-diketones on the respiratory heath of coffee workers. This paper describes the rationale for the exposure and health assessment strategy, the approach used to achieve the study objectives, and its advantages and limitations.

## Introduction

Coffee production is a global industry with roasteries located throughout the world. Production involves receiving green (raw) beans, roasting green beans, grinding roasted beans, in some facilities flavoring roasted ground or whole beans, weighing and packaging roasted and ground, flavored or unflavored coffee, and shipping (1). Workers in this industry are exposed to complex mixtures of gases, dusts, and vapors including carbon monoxide, carbon dioxide, coffee dust, allergens, alpha-diketones, and other VOCs (1). Adverse respiratory health outcomes such as respiratory symptoms, decreased pulmonary function, asthma, and obliterative bronchiolitis (OB), a rare, irreversible lung disease, can occur among exposed workers (2). OB is found among workers exposed to flavoring chemicals in a variety of food processing and flavoring-manufacturing industries (3). Inhalation exposure to alpha-diketones including 2,3-butanedione (diacetyl) and 2,3-pentanedione (acetyl propionyl) in flavorings or natural sources is associated with the development of OB, based on human epidemiologic and animal studies. Mitigating these exposures offers the best opportunity to prevent these adverse respiratory health outcomes (4, 5).

Between 2008 and 2012, two cases of OB were identified in coffee production co-workers exposed to flavoring chemicals (6). Subsequently, three additional cases were diagnosed in former flavoring room workers of the same facility (7). These five cases of OB prompted a request in 2012 to the National Institute for Occupational Safety and Health (NIOSH) to conduct a health hazard evaluation (HHE) to investigate exposures and respiratory effects during coffee production (7, 8). Subsequently, NIOSH received 17 additional HHE requests during 2015–2017 from small- to medium-sized coffee workplaces throughout the United States. HHE requestors expressed concerns about exposure to alpha-diketones and the potential respiratory health effects. The HHE investigations were conducted with a focus on quantifying exposures and adverse respiratory health, evaluating exposure-response relationships and identifying opportunities for exposure mitigation.

HHEs are public health responses to emerging health and safety issues in workplaces mandated by the Occupational Safety and Health Act of 1970 and the Mine Safety and Health Act of 1977, with advantages and some limitations. Their narrowed focus on a particular workplace issue facilitates in-depth investigation that can lead to a resolution of the issue that triggered the investigation, however, the information generated may not always be generalizable knowledge that can be applied more broadly for prevention. Moreover, HHE investigations are constrained by time and resources dedicated to any one investigation. In the coffee industry (North American Industry Classification System code 311920) in 2016, 93% of the establishments were small- (<20 workers) to medium-sized (≥20–<500 workers) workplaces employing 48% of the workforce (9). Individually, these facilities would not have a large enough workforce to provide sufficient power to detect subtle health risks; pooling data across facilities could provide a large enough population to explore exposure-response relationships.

Harmonization of data collection is challenging (10–15) but is required to enable pooling of data for aggregate analysis. A primary challenge is the balance between recording data unique to a particular worksite and collecting data that fit into predetermined standardized categories. With greater standardization and uniformity of data collection, the uniqueness and specificity of each facility may be lost, resulting in exposure or health misclassification, causing loss of any potential gain in statistical power from increase in sample size. There are many examples of successful pooling of data from multiple sources for epidemiologic or exposure studies within an industry, such as the studies in the asphalt and rubber manufacturing industries that pooled exposure data from several European countries (16, 17). However, pooling data across industries is challenging, as highlighted by the numerous calls and proposals over the past three decades for standardization and the development of exposure surveillance databases (18–26); these efforts have failed to gain traction in part because of the complexity and number of data elements to be uniformly collected (27). Within the NIOSH HHE Program, there are some examples of data pooling such as in the microwave popcorn industry where health and exposure data were combined from six plants (28). Additionally, a noise exposure dataset was created by pooling data from 77 HHE reports across various industries, and a dataset of exposure to three solvents, methylene chloride, 1,1,1-trichloroethane, and trichloroethylene was created by pooling data from 63 to 89 HHE and 6-22 Industry Wide Studies reports for the different solvents, which included data elements such as industry, job, hearing protection use, activity, ventilation, and sampling details (29, 30). Some of the variables in the solvents dataset were created after data collection from details in the reports such as the process condition, proximity to source, and ventilation. To ensure systematic collection of contextual information, data elements can be gathered in a tiered approach from more general information collected for all investigations in the first tier that can be pooled across investigations, to more specific information collected in higher tiers targeting nuanced aspects of each facility, and may not always be amenable to pooling, or may be standardized post collection. Such a tiered approach ensures standardized data collection for some basic variables to enable pooling, at the same time enabling the collection of facility specific details to achieve the objectives of the investigation.

## Project Overview and Objectives

NIOSH conducted HHEs at 17 coffee facilities in several geographical locations across the United States during different seasons in 2016–2017. After the HHE investigations were completed and facility-specific reports issued, data from the 17 investigations were pooled to better address HHE requestors' primary concern of whether exposure to alpha-diketones was associated with adverse respiratory effects. At each facility, all workers were invited to participate; 229 (35%) workers participated in the exposure survey and 384 (58%) participated in the health investigation. The analysis of the pooled data was approved by the NIOSH Institutional Review Board (IRB). The specific objectives of the pooled analysis were to (1) quantify full-shift, short-term task-based, and instantaneous exposures to alpha-diketones, (2) identify and quantify factors affecting short-duration and full-shift exposures to alpha-diketones, (3) characterize the respiratory health of workers including pulmonary function and symptomology, (4) evaluate exposure-response relationships with exposure metrics and surrogates such as tasks or proximity to process, and (5) evaluate emission sources and recommend exposure control options.

The objective of this paper is to describe the rationale for the exposure and health assessment strategy, the approach used to achieve the study objectives, and its advantages and limitations.

## Methods and Discussion

### Approach

The investigations focused on characterizing both long-term average exposures and high-intensity, short-duration “peak” exposures to diacetyl and 2,3-pentanedione, because these exposures may be associated with small airways damage related to OB; peak exposures can potentially overwhelm the capacity of normal defense mechanisms and induce adverse health effects (31, 32). Peak exposures to diacetyl have been documented in the microwave popcorn industry and at the sentinel coffee facility and may have contributed to disease in OB cases with relatively lower average exposures (28, 33, 34); the role of peak exposure is asthma is also well recognized (35), though relevant exposure to asthmagens such as green coffee dust and allergens could not be assessed quantitatively due to limited time and resources.

### Exposure Measurement Strategy

To understand personal exposures and characterize emission sources, both personal and area sampling was conducted for alpha-diketones (1). Personal full-shift, short-duration task, and instantaneous peak exposures were collected to better understand their influence on the disease process, while full-shift and instantaneous area samples were collected to identify sources of emissions to prioritize opportunities to control exposures. Instantaneous samples were also analyzed for 18 additional VOCs including: acetaldehyde, acetonitrile, ethanol, isopropyl alcohol, acetone, n-hexane, chloroform, methylene chloride, methyl methacrylate, benzene, ethylbenzene, toluene, styrene, m, p-xylene, o-xylene, a-pinene, and d-limonene. Repeat measurements were collected for all sample types whenever possible to better capture exposure variability. This sampling and analysis approach enabled: (1) characterization of exposure variability through repeated measurements, (2) quantification of multiple alpha-diketones to evaluate their individual and combined effect on respiratory health, (3) development of multiple current or worklife exposure metrics to test different hypotheses of the effect of peak, average, cumulative exposure or exposure duration on health, (4) identification of mixed exposures, albeit limited to VOCs, and their role in the disease process, (5) better understanding of factors affecting exposures by collecting contextual information as described in the next section, and (6) characterization of emission sources to better guide various exposure mitigation strategies. Although the exposure monitoring was comprehensive, it was time and resource intensive to collect five different types of samples, i.e., personal full-shift, short-duration tasks, and instantaneous samples, and area full-shift and instantaneous samples, but necessary to achieve the investigation objectives.

### Exposure Factors, Database Development, and Modeling Exposure Determinants

An integral component of exposure assessment is evaluating exposure variability and understanding factors affecting exposures. Statistical modeling of exposure factors requires the collection of both exposure measurements and detailed contextual information on workplace characteristics such as: processes, control measures, environmental conditions, jobs, tasks, source materials, worker activities, and other relevant work environment factors (18, 36–40). The source-receptor model is a conceptual model that describes the physical pathway of exposure from its generation at the source through different transport compartments and mechanisms to the route of entry at the receptor, and provides a framework to systematically evaluate and collect information on potential micro-level exposure determinants (36–38). At each stage, numerous factors can influence exposure which have been well documented in the literature and should be considered for data collection (36, 40). These within-facility micro-level factors explain differences in exposure caused by tasks or source characteristics. However, differences in exposure can also arise from differences among facility characteristics not directly associated with the physical path of exposure, i.e., higher level factors such as size of the facility, number of workers, production volumes, worker health and safety training, facility safety culture and other organizational factors (37, 38). These higher-tiered factors are particularly important when data are pooled across workplaces or multiple industries. Some factors may be constant within a facility and gathered anytime during the exposure survey (such as general exhaust ventilation parameter), while others may vary in time requiring collection during air sampling (such as tasks, tools, amount of time in different locations) (41). Whereas, attempts to standardize the collection and storage of contextual information have not gained traction (15, 18, 21, 22, 25, 26, 41, 42), numerous studies have successfully collected and used contextual information specific to their research to better understand the causes of variation or to predict exposures (41–43).

In this study, collecting the desired information on exposure determinants during sampling was challenging because of limited time, staff, and resources available to conduct the assessments at each facility. Table 1 presents a list of the key determinants along the source-receptor pathway and some higher-level facility related factors that were collected. Information on facility level determinants which did not vary within a worksite was systematically collected on forms prior to or at some point during the site visit. However, only a handful of time varying factors were gathered during the survey mostly as notes, but which may have the greatest potential for explaining exposure variability within a facility. Determining a priori the time-varying factors to systematically capture during sampling was challenging as there were numerous short-lived activities which would require continuous observation of the monitored workers to capture accurately. The workers performed numerous tasks and were highly mobile making their continuous observation not practicable. Nevertheless, the contextual information collected will be used in multiple regression models of full-shift and task-based diacetyl and 2,3-pentanedione exposure using advanced Bayesian methods that simultaneously account for repeated measures, measurements below the limit of detection, correlation among predictor variables, variable selection for multiple regression modeling and model averaging of multiple final models (44).

**Table 1:** Data elements collected during the 17 HHEs to describe exposures.

**Variables**	**Value and description**
**Facility level information**	
• Region • Total employees • Production employees • Building size • Production ares size • Building type	• US geographical region of facility • Total number of employees on site • Number of production employees on site • Size in square feet • Size in square feet • Production, café, administration
**Source characteristics**	
• Production rate/capacity • Percentage source material • Exposure sources • Open containers • Flavoring	• Amount of material processed per day or capacity • Percent whole bean or ground coffee • Number of sources within 10 ft • Number of open containers of products stored • Flavoring added to coffee beans or ground coffee
**Work organization**	
• Department • Location/work area • Job title • Shift • Task/activity	• Department or work unit • Worker or area sampler location • Worker job title sampled • Work shift and shift hours • Task or activity sampled
**Sampling and analysis**	
• Date • Season • Sample type 1/ 2 • Sampler type • Method • Analyte • Concentration/unit • LOD • Duration	• Sampling date • Sampling season • Area, personal/full-shift, task-based, instantaneous • Silica gel tube, canister • Analytical methods numbers • Analytes quantified, e.g., diacetyl, ethanol, etc. • Concentration value and units • Limit of detection concentration and sample ID • Sample duration or duration of tasks
**Exposure controls**	
• Process isolation • Natural ventilation • GEV • Makeup air • Fans • Machine LEV • Machine enclosure • Automation • Cleaning method • PPE	• Isolated area or process from other areas • Open windows or doors • General exhaust ventilation present and working • Mechanical makeup air • Fans used • Local exhaust ventilation of machines/processes • Enclosure of machines/processes • Automation of machines/processes • Vacuum, dry sweep, compressed air, wet cleaning • Type of respirator used

### Exposure Modules and Job/Task Exposure Matrix

Exposure assessment is a critical component of epidemiologic studies, but can present a significant challenge (45, 46). Many epidemiologic studies use exposure proxies such as job or task exposure matrices (J/TEM), expert judgment, or self-reports (47, 48). Poor exposure characterization can lead to exposure misclassification and attenuation of exposure-response relationships (49) and improper intervention (50). Although personal exposure data are rare in epidemiologic studies, exposure measurements and their determinants collected for jobs or tasks on a subset of workers can be used to create quantitative J/TEM. The J/TEM can be combined with frequency and duration of job or task reported in a questionnaire to calculate exposures for individual study participants (51–53). This approach has been used successfully in several studies (54–56). In situations where job-related exposures are highly variable and depend on tasks performed, a TEM may be preferable (57–59). Under these circumstances, TEMs can result in stronger associations with respiratory health outcomes in different industries compared to JEMs (60–63). Thus, combining worker-specific data (e.g., frequency and duration of tasks performed) from a questionnaire with a J/TEM can result in more accurate estimate of worker exposures compared to a generic J/TEM as it takes into account worker-specific exposure circumstances (64–66). Quantitative exposures are essential to minimize exposure misclassification and obtain quantitative exposure-response relationships to support the development of exposure limits and design of optimal prevention strategies (67).

In this study, information on jobs performed and tenure in the coffee or flavoring industry was gathered in the work history questionnaire. The exposure module elicited information on the frequency and duration of tasks performed in current job, which captures and reflects worker-specific exposure circumstances (Table 2). However, the duration and frequency of tasks performed in previous jobs was not gathered because of the potential for error or bias in recalling such detailed information about tasks in past jobs (68). Full-shift and task-based exposure measurements enabled the construction of JEM and TEM. Furthermore, J/TEM cells included average (the minimum variance unbiased estimator of arithmetic mean) and 95th percentile (P95) job or task exposures as well as measures of peak exposures (P95) from instantaneous sampling. The exposure profiles from multiple tasks and jobs held by workers can be summarized to obtain current or worklife highest, average, and cumulative exposure summary metrics to explore multiple hypotheses about exposure-response relationships. Specifically, these metrics include: the highest instantaneous P95 exposure for current activities; the highest P95 and the average short-duration exposures for current tasks, weighted or unweighted by task duration and frequency; the highest (P95) and average full-shift exposure for current job; and highest (P95), average and cumulative exposures for all jobs as worklife metrics. Worklife metrics based on task-based sampling could not be calculated as historical task information was not gathered. These summary metrics can be calculated for the measured alpha-diketones, as well as their combination as a sum of total alpha-diketones concentration. Additionally, information on the frequency and duration of current tasks can be used as qualitative or quantitative surrogate of total exposure experienced during a task, which includes the combination of alpha-diketones, other VOCs, dusts and allergens depending on the task. The frequency and duration of task information can be combined to obtain the hours per week a task is performed. Performing the task of handling green coffee beans can be a surrogate for exposure to dust and allergens that were not quantified. These wide range of metrics offer the opportunity to fully examine the effects of current or worklife exposure to specific alpha-diketones and their sum, other VOCs, dust, and allergens, peak or cumulative exposure metrics on various respiratory health outcomes of interest ranging from current symptoms to lung function parameters.

**Table 2:** Questions used in the exposure module of the questionnaire to assess exposures.

What is your current job? Job title, tenure years, hours worked/day, days worked/week
What are your past jobs in this facility? Job title, tenure years, hours worked/week, tasks performed
What are your past jobs in other coffee or flavoring facilities? Facility, job title, tenure years
Production area work or by-stander Location, hours in the area/week, production area worker or passer-by
Do you work with green beans?
Do you work in the warehouse or where finished goods are stored? Location of warehouse (on site/off site)
Do you roast coffee beans? Number of days/week, number of hours/day, roaster ID, collect roast sample and smell beans
Do you grind coffee beans? Number of days/week, number of hours/day, grinder ID, grind flavored/unflavored beans
Do you move roasted beans or ground coffee? Number of days/week, number of hours/day
Do you flavor coffee (whole bean or ground coffee)? Number of days/week, number of hours/day, use liquid/powder flavoring, flavor whole bean/ground coffee
Do you package coffee (whole bean or ground coffee)? Number of days/week, number of hours/day, packaging machine/ by hand, machine ID, package machine ID, package flavored/unflavored, repackage faulty packaging
Do you clean containers of roasted coffee? Number of days/week, number of hours/day, clean storage container/roaster/grinder/packaging machine
Do you perform maintenance on coffee production machines? Number of days/week, number of hours/day
Do you perform any quality control activities? QC green beans/roast beans, number of days/week, number of hours/day
Do your grind coffee beans as part of quality control? Number of days/week, number of hours/day, grind flavored/unflavored coffee, flavor coffee
Do you flavor coffee as part of quality control? Number of days/week, number of hours/day, use liquid/powder flavoring, flavor whole bean/ground coffee
Do you perform quality control checks such as brewing, cupping, and tasting? Number of days/week, number of hours/day
Do you work in the café? Number of days/week, number of hours/day
Do you grind coffee beans in the café? Number of days/week, number of hours/day, grind flavored/unflavored beans
Do you flavor brewed coffee in the café? Number of days/week, number of hours/day, use liquid/powder flavoring
Are you ever within an arm's length of these locations/units? Coffee roaster, cooling bins of roasted coffee, grinder, hoppers or containers of roasted coffee, coffee being packaged, packaged coffee, flavoring is being added or mixed

### Health Assessment Strategy

Cross-sectional surveys were conducted to investigate evidence of OB, asthma and other respiratory diseases using a combination of novel and established methods described in detail by Harvey et al. (2). The questionnaire included modules on irritation, upper and lower respiratory symptoms, disease diagnoses, smoking history, work history, and exposure modules; the respiratory health questions were based on validated survey instruments. Spirometry testing was conducted following American Thoracic Society (ATS) guidelines (69) to identify functional respiratory abnormalities, and obstructive, restrictive and mixed pattern were recorded. Impulse oscillometry (IOS) was conducted according to manufacturer instructions and published experience to augment spirometry as a more sensitive metric of small airways dysfunction that may serve as an early indicator by identifying abnormalities in workers with normal spirometry (70, 71). Bronchodilator was administered for those with abnormal spirometry or IOS to evaluate whether these abnormalities were fixed or reversible. Testing for fractional exhaled nitric oxide (FeNO) was conducted following ATS guidelines (72) to identify those with eosinophilic airway inflammation, commonly seen in allergic or Immunoglobulin E mediated asthma.

The health effects of concern in the coffee production facilities include asthma and OB, which may manifest with a range of overlapping respiratory symptoms and functional abnormalities. The objective of the health investigation was to assess the burden of respiratory abnormalities, in particular early markers of disease that could inform prevention. For instance, spirometry provides objective measures of functional abnormalities and their severity, but may be normal in early OB or mild asthma on account of insensitivity to changes in the small airways (73–75). The addition of IOS was thus intended to capture small airways dysfunction that can occur in both OB and asthma and may precede spirometric detection. For those participants with functional abnormalities, performing lung function testing before and after administration of bronchodilator can help identify workers with reversible abnormalities likely related to asthma from those with fixed abnormality likely associated with OB. Test of FeNO may further distinguish those with allergic asthma from those with irritant asthma (72).

IOS is not a new technology (76), but more recent portable units facilitate its use beyond the pulmonary function laboratory, including field studies of the workplace. IOS measures the mechanical properties of the respiratory system including upper and intrathoracic airways, lung tissue and chest wall, specifically respiratory impedance, and is thought to be sensitive for dysfunction of the small airways (77, 78). Histopathological changes in small airways of workers exposed to alpha-diketones are characterized by bronchiolar wall fibrosis, leading to luminal narrowing and obliteration that obstructs airflow (79). A growing body of literature, particularly from the experience with lung transplant patients and survivors of the World Trade Center disaster, indicates that oscillometry can detect this small airway obstruction at an earlier stage than spirometry (73, 80).

Despite these advantages, IOS does pose challenges. Normative values to aid in the interpretation of IOS parameters are not robust and available for only select small sub populations (74, 81). Additionally, little to no research has explored the underlying patterns in the IOS parameters that may identify new markers of early pathophysiologic changes that may be linked to different disease outcomes. There is a potential wealth of information locked in the numerous IOS parameters and flow and volume parameters from spirometry that are not regularly used, such as forced expiratory volume in 3 s (FEV3). If these IOS and spirometry parameters are combined with symptoms, disease diagnoses, demographics, and exposure data to explore patterns, they may provide invaluable insights into early markers of adverse health outcomes (82). Underlying patterns in these complex set of variables can be brought to light by advanced machine learning methods, which may help identify subgroups of workers at different stages of disease development (83), and enable timely intervention to prevent progression of adverse respiratory health outcomes. The combination of these tests along with advanced data analyses will be used to potentially separate those with asthma from those with effects consistent with OB and to potentially identify early markers of adverse health outcomes.

The health assessment strategy, while extensive and thorough, has several limitations with some that are inherent in the nature of HHE mechanism including: (1) inability to assess longitudinal change in symptoms or lung function because of the cross-sectional nature of the study, (2) potential for healthy worker survivor effect because of enrolling current workers only, (3) potential for bias if differential participation by health status occurred as participation was not 100%, and (4) potential for underestimation of exposure and respiratory health burden in the industry as the HHE requests were often made by management at facilities without known health problems. Additionally, despite the extensive respiratory health evaluations, additional medical testing such as collecting blood samples for immune response to allergens and potential novel biomarkers of early pathophysiologic changes, radiographic imaging that may be more sensitive than lung function for small airway diseases and performing challenge testing to assess for airways hyperresponsiveness could result in better characterization of respiratory health.

### Exposure-Response Modeling

A variety of standard and advanced statistical models will be fit for the various outcome measures of interest with the many quantitative exposures to alpha-diketones and quantitative and qualitative metrics of task exposure surrogates. Least-square regression will be used to fit models for continuous outcomes from FeNO, spirometry and IOS parameters. Logistic regression models will be used to model polytomous outcomes from multiple categories of IOS, spirometry, asthma and subgroups identified by machine learning, and binary outcomes from symptoms, chronic disease diagnoses, and other asthma variables. All models will include adjustment for age, sex, race, body mass index, height, weight, smoking status, allergic status, and tenure as appropriate. Model with continuous exposure metrics will be evaluated for non-linearity through fitting restricted cubic splines (84). The various tasks as exposure surrogates are not mutually exclusive thus not independent and cannot be modeled separately or put in the same model due to correlation among tasks. These associations will be explored using advanced statistical methods to account for the effects of multiple correlated exposures and their interactions (85–88).

### Anticipated Outcomes of the Study

The exposure assessment strategy facilitates several exposure metrics to be generated to explore multiple questions on the nature of the exposure-response relationships. The respiratory health outcomes include upper or lower respiratory symptoms, disease diagnoses, spirometry and IOS parameters, and FeNO values. Thus, a spectrum of adverse health effects can be explored, including those that may be related to flavoring chemicals and those that may be related to allergen and irritant exposures such as green coffee bean and dust exposures. The analyses using machine learning methods may identify underlying patterns of various health measures parameters that may represent new markers of early health effects. The various exposure-response relationships can address: (1) the relevance of peak, average, cumulative intensity or duration of exposure, (2) the role of individual or combined alpha-diketones or mixed VOC and dust exposures, and (3) the shape of the exposure-response relationship for the various health outcomes.

Results of exposure modeling can identify the effects of contextual information on full-shift and task-based exposures to enable identification and prioritization of exposure mitigation options. The use of the advanced Bayesian modeling method facilitates the evaluation of all determinants that make it into the numerous (could be as high as one thousand) final models which are then averaged to summarize the importance (% presence in final models) and effect (parameter estimate) of each variable; in traditional modeling, such decisions are made based on a single final model fit using a statistically convenient strategy of forward selection or backward elimination (44). The set of important exposure determinants can inform controls selection by identifying factors with the greatest potential impact on exposure to prioritize.

### Challenges, Limitations and Reflections on the Approach

With any approach, there are trade-offs and limitations of selected study design or strategy. Primary limitations of exposure assessment were: (1) not measuring exposure to dust and allergens that are important for asthma outcomes (though exposure surrogate of the task of handling green beans may provide some insights), (2) difficulty in collecting adequate detail on time-varying contextual information during exposure monitoring, and (3) impracticality of collecting task duration and frequency information for historical jobs. Job titles in the coffee workplaces were often non-specific and did not clearly distinguish among workers and their tasks. Task-based sampling offers the opportunity to directly identify high exposure tasks for targeted controls, may provide more precise estimates of the long-term average exposures for epidemiologic studies under some circumstances when exposures are highly variable (57, 89, 90), can be used to compare calculated full-shift exposure to exposure limits through full or partial period consecutive or grab samples (91), and can provide a range of exposure metrics from average to peak exposure that can be summarized to cumulative, average or highest exposure summary metrics for epidemiologic analysis. Despite some advantages, there are a number of challenges associated with task-based sampling, including task definition, extensive sampling effort, accurately collecting information on the frequency and duration of current and historical tasks, and collecting adequate mass for quantification (68, 92–94). Personal real-time monitoring of alpha-diketones and other VOCs would have been ideal, but currently available technology, e.g., a portable Fourier Transform Infrared Spectrometer is an area sampler not suited for personal monitoring (95). With advances in sensor technologies, real-time wearable sensors may be available in the future to capture instantaneous, short-duration and full-shift exposures with one sampler to greatly reduce monitoring effort.

Collecting contextual information is challenging as it entails accounting for time over which innumerable factors may change causing measured exposures to vary considerably. Collecting time-varying contextual information requires direct observation or worker-recording of tasks, duration or frequency, and other factors along the source-receptor pathway, which is challenging and time and resource intensive (56, 96–98). Factors that are constant within a facility and do not vary over time are easier to collect but may explain limited within-facility variability. Contextual variables for task-based samples taken over a shorter period can be short lived and highly variable, and may be best recorded as present or absent to minimize error in estimating duration. New sensor technologies may make it easier to capture contextual information in real-time more accurately without having to continuously observe workers (99). For example, the Dutch Institution of Applied Science, TNO is piloting the use of sensors to detect and record proximity of workers to different sources of exposure, or placing sensors on tools or machines to record their operational information such as vibration sensors on tools or machines to indicate when in use.

While the health assessment was extensive, a comprehensive health assessment is time and resource prohibitive and not practicable for HHEs. Additionally, some potential biases could have been minimized by including former workers and recruiting additional worksites with potentially more varied burden of respiratory disease and exposure experiences, but this was not feasible. Likewise, longitudinal follow-up study design would be ideal but not typical within the HHE Program context. Nevertheless, the combination of several tests with advanced machine learning methods holds promise for the potential identification of early markers of respiratory effects.

## Conclusion

The overall goal of the study was to characterize exposure conditions, respiratory health, and exposure-response relationships among coffee production workers, ultimately leading to exposure mitigation and prevention of adverse respiratory health outcomes. To achieve these goals, extensive exposure and health assessments were conducted within the confines of the HHE Program. Data were pooled to provide a large enough population to explore exposure-response relationships to address one of the requestors' primary concerns about the effect of exposure to alpha-diketones on the respiratory heath of coffee workers. Strengths and limitations of the approaches used are discussed. It must also be emphasized that when use of secondary data from individual HHEs is deemed to constitute human subjects research, all regulations governing human subjects research must be followed including approval from the NIOSH IRB.

## Data Availability Statement

The original contributions presented in the study are included in the article/supplementary material, further inquiries can be directed to the corresponding author.

## Author Contributions

MV, KC, and JC-G contributed conception and design of the study. MV wrote the first draft of the manuscript. All authors contributed to manuscript revision, read, and approved the submitted version.

## Funding

This work was supported by the National Institute for Occupational Safety and Health (NIOSH).

## Author Disclaimer

The findings and conclusions in this report are those of the authors and do not necessarily represent the official position of the National Institute for Occupational Safety and Health, Centers for Disease Control and Prevention.

## Conflict of Interest

The authors declare that the research was conducted in the absence of any commercial or financial relationships that could be construed as a potential conflict of interest.

## Publisher's Note

All claims expressed in this article are solely those of the authors and do not necessarily represent those of their affiliated organizations, or those of the publisher, the editors and the reviewers. Any product that may be evaluated in this article, or claim that may be made by its manufacturer, is not guaranteed or endorsed by the publisher.

## References

[1] LeBouf RF Blackley BH Fortner AR Stanton M Martin SB Groth CP. Exposures and emissions in coffee roasting facilities and cafés: diacetyl, 2,3-pentanedione, and other volatile organic compounds. Front public health. (2020) 8:561740. 10.3389/fpubh.2020.561740 33072698 PMC7531227

[2] Harvey RR Fechter-Leggett ED Bailey RL Edwards NT Fedan KB Virji MA. The burden of respiratory abnormalities among workers at coffee roasting and packaging facilities. Front public health. (2020) 8:5. 10.3389/fpubh.2020.00005 32083049 PMC7003510

[3] McKernan LT Niemeier RT Kreiss K Hubbs Ann F Park R Dankovic DA. Occupational Exposure to Diacetyl And 2,3-Pentanedione. Cincinnati, OH: U.S. Department of Health and Human Services, Centers for Disease Control and Prevention, National Institute for Occupational Safety and Health, DHHS (NIOSH) Publication No. 2016-111 (2016).

[4] Dunn KH McKernan LT Garcia A. Best Practices: Engineering Controls, Work Practices, and Exposure Monitoring For Occupational Exposures to Diacetyl And 2, 3-Pentanedione. Cincinnati, OH: U.S. Department of Health and Human Services, Centers for Disease Control and Prevention, National Institute for Occupational Safety and Health, DHHS (NIOSH) Publication 2015–197 (2015).

[5] Stanton ML McClelland TLBM Ranpara A Martin SB. Efficacy of engineering controls in mitigating diacetyl and 2,3-pentanedione emissions during coffee grinding. Front public health. (2021). 10.3389/fpubh.2022.750289 PMC9159804 35664098

[6] Huff S Stocks JM Saito R Bilhartz P Levin J Glazer C. Obliterative bronchiolitis in workers in a coffee-processing facility—Texas, 2008–2012. MMWR Morb Mortal Wkly Rep. (2013) 62:305. 23615673 PMC4604960

[7] Bailey RL Cox-Ganser JM Duling MG LeBouf RF Martin SB Jr. Bledsoe TA. Respiratory morbidity in a coffee processing workplace with sentinel obliterative bronchiolitis cases. Am J Ind Med. (2015) 58:1235–45. 10.1002/ajim.22533 26523478 PMC4715657

[8] NIOSH. NIOSH Health Hazard Evaluations (HHE) (2021). Available online at: https://www.cdc.gov/niosh/hhe/

[9] USCB. Statistics of U.S. Businesses (SUSB) Annual Data Tables by Establishment Industry (2016). Available online at: https://www.census.gov/data/tables/2016/econ/susb/2016-susb-annual.htm

[10] Davies H. Capacity Development for a Canadian Workplace Exposure Database. Report to the WorkSafeBC Research Services “FOCUS ON TOMORROW “RESEARCH AT WORK” program. Report number: RS-2010_OG13 (2014).

[11] Lavoue J Friesen M Burstyn I. Workplace measurements by the US Occupational Safety and Health Administration since 1979: descriptive analysis and potential uses for exposure assessment. Ann Occup Hyg. (2013) 57:77–97. 10.1093/annhyg/met027 22952385 PMC3589950

[12] Mater G Paris C Lavoué J. Descriptive analysis and comparison of two French occupational exposure databases: COLCHIC and SCOLA. Am J Ind Med. (2016) 59:379–91. 10.1002/ajim.22569 26901238

[13] Stamm R. MEGA-database: one million data since (1972). Appl Occup Environ Hyg. (2001) 16:159–63. 10.1080/104732201460262 11217704

[14] Vincent R Jeandel B. COLCHIC - occupational exposure to chemical agents database: current content and development perspectives. Appl Occup Environ Hyg. (2001) 16:115–21. 10.1080/104732201460190 11217697

[15] Brederode D Linker F Marquart H Pothuis J Slijpen J Timmermans H. Recording of data of individual measurements of occupational exposure: guideline of the Dutch Society of Occupational Hygiene (October 1999). Appl Occup Environ Hyg. (2001) 16:122–7. 10.1080/104732201460208 11217698

[16] Burstyn I Kromhout H Cruise PJ Brennan P. Designing an international industrial hygiene database of exposures among workers in the asphalt industry. Ann Occup Hyg. (2000) 44:57–66. 10.1016/S0003-4878(99)00102-7 10689759

[17] De Vocht F Straif K Szeszenia-Dabrowska N Hagmar L Sorahan T Burstyn I. A database of exposures in the rubber manufacturing industry: design and quality control. Ann Occup Hyg. (2005) 49:691–701. 10.1093/annhyg/mei035 16126766

[18] Lippmann M Gomez MR Rawls GM. Data elements for occupational exposure databases: guidelines and recommendations for airborne hazards and noise. Appl Occup Environ Hyg. (1996) 11:1294–311. 10.1080/1047322X.1996.10389417

[19] Rajan B Alesbury R Carton B Gérin M Litske H Marquart H. European proposal for core information for the storage and exchange of workplace exposure measurements on chemical agents. Appl Occup Environ Hyg. (1997) 12:31–9. 10.1080/1047322X.1997.10389453

[20] Boiano JM Hull RD. Development of a national occupational exposure survey and database associated with niosh hazard surveillance initiatives. Appl Occup Environ Hyg. (2001) 16:128–34. 10.1080/104732201460217 11217699

[21] Gómez MR. Recommendations for optimizing the usefulness of existing exposure databases for public health applications. AIHA J. (1997) 58:181. 10.1080/00028894.1997.10399236

[22] LaMontagne AD Herrick RF Dyke MVV Martyny JW Ruttenber AJ. Exposure databases and exposure surveillance: promise and practice. AIHA J. (2002) 63:205–12. 10.1080/15428110208984706 11975658

[23] Melville R Lippmann M. Influence of data elements in OSHA air sampling database on occupational exposure levels. Appl Occup Environ Hyg. (2001) 16:884–99. 10.1080/10473220117850

[24] Morgan DA. Occupational exposure databases and their application for the next millennium: symposium framework and workshop introduction. In: Applied Occupational and Environmental Hygiene. Taylor & Francis Group (2001). 10.1080/104732201460181

[25] Ritchie PJ Cherrie JW. The development of a prototype database for the voluntary reporting of occupational exposure data on chemicals. Appl Occup Environ Hyg. (2001) 16:295–9. 10.1080/10473220118921 11217726

[26] Stewart P Stenzel M. Data needs for occupational epidemiologic studies. J environ monit. (1999) 1:75n−82n. 10.1039/a903405f 11529156

[27] Shockle TM Dahm MM Wurzelbacher SJ Baker J. Industrial hygiene data standardization: past lessons, present challenges, and future directions. Synergist. (2020)

[28] Kanwal R Kullman G Piacitelli C Boylstein R Sahakian N Martin S. Evaluation of flavorings-related lung disease risk at six microwave popcorn plants. J Occup Environ Med. (2006) 48:149–57. 10.1097/01.jom.0000194152.48728.fb 16474263

[29] NIOSH. NIOSH Health Hazard Evaluation Program Noise Measurement Database (2014). Available online at: https://www.cdc.gov/niosh/data/datasets/rd-1005-2014-0/default.html.

[30] Hein MJ Waters MA Ruder AM Stenzel MR Blair A Stewart PA. Statistical modeling of occupational chlorinated solvent exposures for case-control studies using a literature-based database. Ann Occup Hyg. (2010) 54:459–72. 10.1093/annhyg/meq027 20418277 PMC2913720

[31] Kriebel D Checkoway H Pearce N. Exposure and dose modelling in occupational epidemiology. Occup Environ Med. (2007) 64:492–8. 10.1136/oem.2006.030031 17582090 PMC2078465

[32] Smith TJ. Studying peak exposure - toxicology and exposure statistics. In: Marklund S editor. X2001 Exposure Assessment in Epidemiology and Practice. Stockholm: National Institute for Working Life (2001). p. 207-9.

[33] Martyny JW Van Dyke MV Arbuckle S Towle M Rose CS. Diacetyl exposures in the flavor manufacturing industry. J Occup Environ Hyg. (2008) 5:679–88. 10.1080/15459620802368463 18720288

[34] Duling MG LeBouf RF Cox-Ganser JM Kreiss K Martin SB Jr. Environmental characterization of a coffee processing workplace with obliterative bronchiolitis in former workers. J Occup Environ Hyg. (2016) 13:770–81. 10.1080/15459624.2016.1177649 27105025 PMC5836548

[35] Virji MA Kurth L. Peak inhalation exposure metrics used in occupational epidemiologic and exposure studies. Front public health. (2020) 8:611693. 10.3389/fpubh.2020.611693 33490023 PMC7820770

[36] Tielemans E Schneider T Goede H Tischer M Warren N Kromhout H. Conceptual model for assessment of inhalation exposure: defining modifying factors. Ann Occup Hyg. (2008) 52:577–86. 10.1093/annhyg/men059 18787181

[37] Virji M Abbas A. Thomas statistical models of exposure determinants. In: Charles B Keil CES editor. Mathematical models for estimating occupational exposure to chemicals. Second ed. Fairfax, VA: AIHA (2009).

[38] Woskie SR Bello D Virji MA Stefaniak AB. Understanding workplace processes and factors that influence exposures to engineered nanomaterials. Int J Occup Environ Health. (2010) 16:365–77. 10.1179/oeh.2010.16.4.365 21222381

[39] Cherrie JW Fransman W Heussen GAH Koppisch D Jensen KA. Exposure models for REACH and occupational safety and health regulations. Int J Environ Res Public Health. (2020) 17:383. 10.3390/ijerph17020383 31936022 PMC7013818

[40] Marquart H Schneider T Goede H Tischer M Schinkel J Warren N. Classification of occupational activities for assessment of inhalation exposure. Ann Occup Hyg. (2011) 55:989–1005. 10.1093/annhyg/mer072 21926067

[41] Burstyn I Teschke K. Studying the determinants of exposure: a review of methods. Am Ind Hyg Assoc J. (1999) 60:57–72. 10.1080/00028899908984423 10028617

[42] Schinkel J Ritchie P Goede H Fransman W Van Tongeren M Cherrie JW. The Advanced REACH Tool (ART): incorporation of an exposure measurement database. Annals of occupational hygiene. (2013) 57:717–27. 10.1093/annhyg/mes103 23307863

[43] Davies HW Gorman-Ng M. Development of a web-based tool for risk assessment and exposure control planning of silica-producing tasks in the construction sector. Front public health. (2020) 8. 10.3389/fpubh.2020.00371 32850594 PMC7419426

[44] Lavoué J Droz PO. Multimodel inference and multimodel averaging in empirical modeling of occupational exposure levels. Ann Occup Hyg. (2009) 53:173–80. 10.1093/annhyg/men085 19174483

[45] Breysse PN editor. Chemical and Biological Sensing Needs for Health Effects Studies. SPIE Defense, Security, and Sensing; International Society for Optics and Photonics (2012).

[46] Heederik D van Rooy F. Exposure assessment should be integrated in studies on the prevention and management of occupational asthma. Occup Environ Med. (2008) 65:149–50. 10.1136/oem.2005.024711 18283124

[47] Friesen MC LJ Teschke K van Tongeren M. Occupational exposure asssessment in industry and populationa based epidemiologic studies. In: Nieuwenhuijsen MJ editor. Exposure Assessment In Environmental Epidemiology. USA: Oxford University Press (2015) p. 139-62.

[48] Teschke K Olshan A Daniels J De Roos A Parks C Schulz M. Occupational exposure assessment in case–control studies: opportunities for improvement. Occup Environ Med. (2002) 59:575–94. 10.1136/oem.59.9.575 12205230 PMC1740358

[49] Armstrong B. Exposure measurement error: consequences and design issues. In: Nieuwenhuijsen BW editor. Exposure Assessment in Occupational and Environmental Epidemiology. New York, NY: Oxford University Press (2003).

[50] Heederik D. Cleaning agents and disinfectants: moving from recognition to action and prevention. Clin Exp Allergy. (2014) 44:472–4. 10.1111/cea.12286 24666520

[51] Coble JB Lees PS Matanoski G. Time trends in exposure measurements from OSHA compliance inspections of the pulp and paper industry. Appl Occup Environ Hyg. (2001) 16:263–70. 10.1080/10473220120763 11217721

[52] Lavoue J Vincent R Gerin M. Formaldehyde exposure in U.S. industries from OSHA air sampling data. J Occup Environ Hyg. (2008) 5:575–87. 10.1080/15459620802275023 18618336

[53] Teschke K Marion SA Vaughan TL Morgan MS Camp J. Exposures to wood dust in U.S. industries and occupations, 1979 to (1997). Am J Ind Med. (1999) 35:581-9. 10.1002/(sici)1097-0274(199906)35:6<581::aid-ajim5>3.0.co;2-i 10332511

[54] Burstyn I Kromhout H Kauppinen T Heikkila P Boffetta P. Statistical modelling of the determinants of historical exposure to bitumen and polycyclic aromatic hydrocarbons among paving workers. Ann Occup Hyg. (2000) 44:43–56. 10.1016/S0003-4878(99)00101-5 10689758

[55] Hornung RW Greife AL Stayner LT Steenland NK Herrick RF Elliott LJ. Statistical model for prediction of retrospective exposure to ethylene oxide in an occupational mortality study. Am J Ind Med. (1994) 25:825–36. 10.1002/ajim.4700250607 8067360

[56] Preller L Kromhout H Heederik D Tielen MJ. Modeling long-term average exposure in occupational exposure-response analysis. Scand J Work Environ Health. (1995) 21:504–12. 10.5271/sjweh.67 8824757

[57] Benke G Sim M Fritschi L Aldred G. Beyond the job exposure matrix (JEM): the task exposure matrix (TEM). Ann Occup Hyg. (2000) 44:475–82. 10.1016/S0003-4878(00)00004-1 10963712

[58] Benke G Sim M Fritschi L Aldred G. A task exposure database for use in the alumina and primary aluminium industry. Appl Occup Environ Hyg. (2001) 16:149–53. 10.1080/104732201460244 11217702

[59] Fevotte J Charbotel B Muller-Beauté P Martin JL Hours M Bergeret A. Case-control study on renal cell cancer and occupational exposure to trichloroethylene. Part I: Exposure assessment. Ann Occup Hyg. (2006) 50:765–75. 10.1093/annhyg/mel039 16840434

[60] Fritschi L Sim MR Forbes A Abramson MJ Benke G Musk AW. Respiratory symptoms and lung-function changes with exposure to five substances in aluminium smelters. Int Arch Occup Environ Health. (2003) 76:103–10. 10.1007/s00420-002-0398-1 12733082

[61] Beach JR de Klerk NH Fritschi L Sim MR Musk AW Benke G. Respiratory symptoms and lung function in bauxite miners. Int Arch Occup Environ Health. (2001) 74:489–94. 10.1007/s004200100245 11697452

[62] Pesch B Haerting J Ranft U Klimpel A Oelschlägel B Schill W. Occupational risk factors for renal cell carcinoma: agent-specific results from a case-control study in Germany. MURC Study Group. Multicenter urothelial and renal cancer study. Int J Epidemiol. (2000) 29:1014–24. 10.1093/ije/29.6.1014 11101542

[63] Woskie SR Bello D Gore RJ Stowe MH Eisen EA Liu Y. Comparison of task-based exposure metrics for an epidemiologic study of isocyanate inhalation exposures among autobody shop workers. J Occup Environ Hyg. (2008) 5:588–98. 10.1080/15459620802275429 18615291

[64] Kromhout H Vermeulen R. Application of job-exposure matrices in studies of the general population-some clues to their performance. Eur Respir Rev. (2001) 11:80–90.

[65] Semple SE Dick F Cherrie JW. Exposure assessment for a population-based case-control study combining a job-exposure matrix with interview data. Scand J Work Environ Health. (2004) 30:241–8. 10.5271/sjweh.785 15250653

[66] Kromhout H Symanski E Rappaport SM. A comprehensive evaluation of within- and between-worker components of occupational exposure to chemical agents. Ann Occup Hyg. (1993) 37:253–70. 8346874 10.1093/annhyg/37.3.253

[67] Becklake M Malo J Chan Yeung M. Epidemiological Approaches to Occupational Asthma. In: Bernstein I Malo J Bernstein D Chan Yeung M editors. Asthma in the Workplace. Third Edition ed. New York, NY: Taylor and Francis Group (2006).

[68] Pulido JA Barrero LH Mathiassen SE Dennerlein JT. Correctness of self-reported task durations: a systematic review. Ann Work Expo Health. (2017) 62:1–16. 10.1093/annweh/wxx094 29228093

[69] Miller MR Hankinson J Brusasco V Burgos F Casaburi R Coates A. Standardisation of spirometry. Eur Respir J. (2005) 26:319–38. 10.1183/09031936.05.00034805 16055882

[70] Berger KI Reibman J Oppenheimer BW Vlahos I Harrison D Goldring RM. Lessons from the World Trade Center disaster: airway disease presenting as restrictive dysfunction. Chest. (2013) 144:249–57. 10.1378/chest.12-1411 23392588 PMC3707176

[71] Jordan HT Friedman SM Reibman J Goldring RM Miller Archie SA Ortega F. Risk factors for persistence of lower respiratory symptoms among community members exposed to the (2001). World Trade Center terrorist attacks. Occup Environ Med. (2017) 74:449–55. 10.1136/oemed-2016-104157 28341697 PMC5520238

[72] Dweik RA Boggs PB Erzurum SC Irvin CG Leigh MW Lundberg JO. An official ATS clinical practice guideline: interpretation of exhaled nitric oxide levels (FENO) for clinical applications. Am J Respir Crit Care Med. (2011) 184:602–15. 10.1164/rccm.9120-11ST 21885636 PMC4408724

[73] Berger KI Turetz M Liu M Shao Y Kazeros A Parsia S. Oscillometry complements spirometry in evaluation of subjects following toxic inhalation. ERJ Open Research. (2015) 1:00043-2015. 10.1183/23120541.00043-2015 27730155 PMC5005120

[74] Berger KI Wohlleber M Goldring RM Reibman J Farfel MR Friedman SM. Respiratory impedance measured using impulse oscillometry in a healthy urban population. ERJ Open Res. (2021) 7:00560-2020. 10.1183/23120541.00560-2020 33816605 PMC8005688

[75] Usmani OS Han MK Kaminsky DA Hogg J Hjoberg J Patel N. Seven pillars of small airways disease in asthma and COPD–Supporting opportunities for novel therapies. Chest. (2021) 160:114–34. 10.1016/j.chest.2021.03.047 33819471

[76] DuBois AB Brody AW Lewis DH Burgess BF Jr. Oscillation mechanics of lungs and chest in man. J Appl Physiol. (1956) 8:587–94. 10.1152/jappl.1956.8.6.587 13331841

[77] King GG Bates J Berger KI Calverley P de Melo PL Dellacà RL. Technical standards for respiratory oscillometry. Eur Respir J. (2020) 55:1900753. 10.1183/13993003.00753-2019 31772002

[78] Cottini M Licini A Lombardi C Bagnasco D Comberiati P Berti A. Small airway dysfunction and poor asthma control: a dangerous liaison. Clin Mol Allergy. (2021) 19:7. 10.1186/s12948-021-00147-8 34051816 PMC8164746

[79] Hubbs AF Kreiss K Cummings KJ Fluharty KL O'Connell R Cole A. Flavorings-Related lung disease: a brief review and new mechanistic data. Toxicol Pathol. (2019) 47:1012–26. 10.1177/0192623319879906 31645208

[80] Cho E Wu JK Birriel DC Matelski J Nadj R DeHaas E. Airway oscillometry detects spirometric-silent episodes of acute cellular rejection. Am J Respir Crit. (2020) 201:1536–44. 10.1164/rccm.201908-1539OC 32135068

[81] Kalchiem-Dekel O Hines SE. Forty years of reference values for respiratory system impedance in adults: 1977-2017. Respir Med. (2018) 136:37–47. 10.1016/j.rmed.2018.01.015 29501245

[82] Hoesterey D Das N Janssens W Buhr RG Martinez FJ Cooper CB. Spirometric indices of early airflow impairment in individuals at risk of developing COPD: spirometry beyond FEV(1)/FVC. Respir Med. (2019) 156:58–68. 10.1016/j.rmed.2019.08.004 31437649 PMC6768077

[83] Giri PC Chowdhury AM Bedoya A Chen H Lee HS Lee P. Application of machine learning in pulmonary function assessment where are we now and where are we going? Front Physiol. (2021) 12:678540. 10.3389/fphys.2021.678540 34248665 PMC8264499

[84] Desquilbet L Mariotti F. Dose-response analyses using restricted cubic spline functions in public health research. Stat Med. (2010) 29:1037–57. 10.1002/sim.3841 20087875

[85] Taylor KW Joubert BR Braun JM Dilworth C Gennings C Hauser R. Statistical approaches for assessing health effects of environmental chemical mixtures in epidemiology: lessons from an innovative workshop. Environ Health Perspect. (2016) 124:A227–a9. 10.1289/EHP547 27905274 PMC5132642

[86] Stafoggia M Breitner S Hampel R Basagana X. Statistical approaches to address multi-pollutant mixtures and multiple exposures: the state of the science. Curr Environ Health Rep. (2017) 4:481–90. 10.1007/s40572-017-0162-z 28988291

[87] Lenters V Vermeulen R Portengen L. Performance of variable selection methods for assessing the health effects of correlated exposures in case-control studies. Occup Environ Med. (2018) 75:522–9. 10.1136/oemed-2016-104231 28947495

[88] Bobb JF Claus Henn B Valeri L Coull BA. Statistical software for analyzing the health effects of multiple concurrent exposures *via* Bayesian kernel machine regression. Environ Health. (2018) 17:67. 10.1186/s12940-018-0413-y 30126431 PMC6102907

[89] Nicas M Spear RC. A task-based statistical model of a worker's exposure distribution: Part I–Description of the model. Am Ind Hyg Assoc J. (1993) 54:211–20. 10.1080/15298669391354586 8498356

[90] Nicas M Spear RC. A task-based statistical model of a worker's exposure distribution: Part II–Application to sampling strategy. Am Ind Hyg Assoc J. (1993) 54:221–7. 10.1080/15298669391354595 8498357

[91] Leidel N Busch K Lynch J. Occupational Exposure Sampling Strategy Manual (OESSM). Palo Alto, CA: NIOSH (1977).

[92] Goldberg M Levin SM Doucette JT Griffin G. A task-based approach to assessing lead exposure among iron workers engaged in bridge rehabilitation. Am J Ind Med. (1997) 31:310–8. 9055954 10.1002/(sici)1097-0274(199703)31:3<310::aid-ajim7>3.0.co;2-0

[93] Virji MA Woskie SR Pepper LD. Task-based lead exposures and work site characteristics of bridge surface preparation and painting contractors. J Occup Environ Hyg. (2009) 6:99–112. 10.1080/15459620802615772 19065390

[94] Virji MA Woskie SR Waters M Brueck S Stancescu D Gore R. Agreement between task-based estimates of the full-shift noise exposure and the full-shift noise dosimetry. Ann Occup Hyg. (2009) 53:201–14. 10.1093/annhyg/mep010 19282390

[95] McCoy MJ Hoppe Parr KA Anderson KE Cornish J Haapala M Greivell J. Diacetyl and 2,3-pentanedione in breathing zone and area air during large-scale commercial coffee roasting, blending and grinding processes. Toxicol Rep. (2017) 4:113–22. 10.1016/j.toxrep.2017.01.004 28959632 PMC5615090

[96] Hansen DJ Whitehead LW. The influence of task and location on solvent exposures in a printing plant. Am Ind Hyg Assoc J. (1988) 49:259–65. 3400590 10.1080/15298668891379710

[97] Kalil AJ Woskie SR Holcroft C Ellenbecker M Buchholz B. Time variant exposure analysis (TVEA): a measurement tool for characterizing particulate exposure determinants in construction. J Occup Environ Hyg. (2004) 1:816–25. 10.1080/15459620490889208 15742711

[98] Ross AS Teschke K Brauer M Kennedy SM. Determinants of exposure to metalworking fluid aerosol in small machine shops. Ann Occup Hyg. (2004) 48:383–91. 15240341 10.1093/annhyg/meh042

[99] le Feber M Jadoenathmisier T Goede H Kuijpers E Pronk A. Ethics and privacy considerations before deploying sensor technologies for exposure assessment in the workplace: results of a structured discussion amongst Dutch stakeholders. Ann Work Expo Health. (2021) 65:3–10. 10.1093/annweh/wxaa093 33057665

